# Changes in Brain Matrix Glycan Sulfation Associate With Reactive Gliosis and Motor Coordination in Mice With Head Trauma

**DOI:** 10.3389/fnbeh.2021.745288

**Published:** 2021-10-28

**Authors:** Kimberly M. Alonge, Melanie J. Herbert, Mayumi Yagi, David G. Cook, William A. Banks, Aric F. Logsdon

**Affiliations:** ^1^Department of Medicine, University of Washington Medicine Diabetes Institute, University of Washington, Seattle, WA, United States; ^2^Geriatric Research Education and Clinical Center (GRECC), Veterans Affairs Puget Sound Health Care System, Seattle, WA, United States; ^3^Division of Gerontology and Geriatric Medicine, Department of Medicine, University of Washington, Seattle, WA, United States

**Keywords:** traumatic brain injury, extracellular matrix, glycosaminoglycans (GAGs), chondroitin sulfate (CS), perineuronal nets (PNNs)

## Abstract

Perineuronal nets (PNNs) are extracellular matrix (ECM) structures that enmesh and regulate neurocircuits involved in motor and sensory function. Maladaptive changes to the composition and/or abundance of PNNs have been implicated in preclinical models of neuroinflammation and neurocircuit destabilization. The central nervous system (CNS) is limited in its capacity to repair and reorganize neural networks following traumatic brain injury (TBI) and little is known about mechanisms of ECM repair in the adult brain after TBI. In this study, adult male C57BL/6 mice were subjected to a TBI *via* a controlled cortical impact (CCI) to the right motor and somatosensory cortices. At 7 days following CCI, histological analysis revealed a loss of *Wisteria floribunda* agglutinin (WFA) positive PNN matrices in the ipsilateral cortex. PNNs are comprised of chondroitin sulfate (CS) and dermatan sulfate (DS)-glycosaminoglycans (GAGs), the composition of which are known to influence neuronal integrity and repair. Using an innovative liquid chromatography tandem mass spectrometry (LC-MS/MS) method, we analyzed the relative abundance of six specific CS/DS-GAG isomers (Δ4S-, Δ6S-, Δ4S6S-, Δ2S6S-, Δ0S-CS, and Δ2S4S-DS) from fixed-brain sections after CCI injury. We report a significant shift in CS/DS-GAG sulfation patterns within the rostro-caudal extent of the injury site from mice exposed to CCI at 7 days, but not at 1 day, post-CCI. In the ipsilateral thalamus, the appearance of WFA^+^ puncta occurred in tandem with gliosis at 7 days post-CCI, but weakly colocalized with markers of gliosis. Thalamic WFA^+^ puncta showed moderate colocalization with neuronal ubiquitin C-terminal hydrolase L1 (UCHL1), a clinical biomarker for TBI injury. A shift in CS/DS-GAG sulfation was also present in the thalamus including an increase of 6S-CS, which is a specific isomer that associates with the presence of glial scarring. Upregulation of the 6S-CS-specific sulfotransferase (CHST3) gene expression was accompanied by reactive gliosis in both the ipsilateral cortex and thalamus. Moreover, changes in 6S-CS extracted from the thalamus positively correlated with deficits in motor coordination after CCI. Collectively, these data argue that CCI alters CS/DS-GAG sulfation in association with the spatiotemporal progression of neurorepair. Therapeutic interventions targeting restoration of CS/DS-GAG sulfation patterns may improve outcomes from TBI.

## Introduction

Perineuronal nets (PNNs) are specialized extracellular matrix (ECM) lattices that enmesh neurons and serve as key regulators of neurocircuit plasticity and activity (Pizzorusso et al., 2002; Carulli et al., 2010; Kwok et al., 2011; Miyata et al., 2012). Comprised of chondroitin sulfate and dermatan sulfate glycosaminoglycan (CS/DS-GAG) chains attached to CS proteoglycan (CSPG) core proteins, the biological function of PNNs are mainly influenced by the sulfate modifications of the CS/DS glucuronic acid (GlcA)-*N*-acetylgalactosamine (GalNAc; CS) and iduronic acid (IdoA)-GalNAc (DS) disaccharide units (Kwok et al., 2011). In the central nervous system (CNS), there exists six biologically relevant CS/DS-GAG isomers (4S-, 6S-, 2S6S-, 4S6S-, 0S-CS and 2S4S-DS; Alonge et al., 2019), the relative abundance of which influence neurological processes including protein-glycan interactions (DS-CS; Djerbal et al., 2017; Nadanaka et al., 2020), PNN stability (4S-CS; Miyata et al., 2012), gliosis after CNS injury (6S-CS; Properzi et al., 2005), and neurite extension inhibition (4S6S-CS; Brown et al., 2012), and extension attraction (2S6S-CS; Shida et al., 2019). CS/DS-GAG sulfation occurs through a variety of chondroitin sulfotransferases (CST) in the endoplasmic reticulum, and the resulting specific CS/DS-GAG sulfation patterns greatly influence CS/DS-GAG interactions with extracellular signaling molecules (Djerbal et al., 2017).

Reports indicate that traumatic brain injury (TBI) increases selective CST expression and alters CS/DS-GAG sulfation patterns in the rat brain, although the alteration of these behavioral outcomes is currently unclear (Properzi et al., 2005). Preclinical models of TBI induce maladaptive ECM/PNN reorganization that associates with the formation of glial scarring in the CNS (Properzi et al., 2005; Lin et al., 2008; Yi et al., 2012). Glial scar formation by astrocytes and other glial cell types is driven by the secretion of specific CSPGs and their associated sulfated CS/DS-GAG chains into the ECM (Silver and Miller, 2004). Specifically, reactive astrocytes and microglia release inflammatory CSPGs (neurocan, brevican, phosphacan) under conditions of CNS inflammation, and the production of these glial-scar CS/DS-GAGs are thought to inhibit axon regeneration (Bradbury et al., 2002). Paradoxically, transgenic loss-of-function experiments to prevent astroglial scarring also impair axon regeneration in mouse models of CNS injury (Anderson et al., 2016). These diverging results imply that the glial scar may both inhibit and promote CNS axon regeneration depending perhaps on the spatiotemporal distribution of the glial scar, composition, and injury severity. Glial marker glial fibrillary acidic protein (GFAP) and neuronal ubiquitin C-terminal hydrolase L1 (UCHL1) are both CSF and blood biomarkers of brain injury in patients with TBI (Brophy et al., 2011; Wang et al., 2021). Here, we explore whether markers of glial scarring and neuronal injury contribute to changes in ECM/PNN GAG composition after CCI.

Little is known regarding the spatiotemporal profile of CS/DS-GAG recoding in relation to neuroinflammation and neurorepair in TBI adult brain tissue. As such, injury-related shifts in CST expression (Properzi et al., 2005) and downstream CS/DS-GAG sulfation pattern reorganizations may play a vital role in the control of neurological function (Carulli et al., 2010). For example, overexpression of 6S-CS sulfotransferase (CHST3) promotes prolonged neurocircuit plasticity and represses the inhibitory firing properties of the underlying neurons (Miyata et al., 2012), and mice deficient in CHST3 exhibit restrained plasticity and limited axon regeneration (Lin et al., 2011). Here, we asked whether mice exposed to CCI demonstrate shifts in CS/DS-GAG sulfation patterns in association with the onset and severity of gliosis and motor impairment in this model of TBI. By extracting and quantitatively analyzing CS/DS-GAG sulfation patterns from brain regions introduced to CCI-induced glial scar formation, we showed TBI induces distinctive changes in the brain CS/DS-GAG composition that associate with the spatiotemporal development of glial scarring, neuronal inclusions, and motor deficits.

## Material and Methods

### Animals

Male C57BL/6 (*n* = 40) mice (Jackson Laboratories) 8–12 weeks of age were studied. Mice had *ad libitum* access to food, water, and were group-housed. Animals were randomly assigned to Naïve, Sham, or CCI groups. All studies were approved by the Veterans Affairs Puget Sound Health Care System’s Institutional Animal Care and Use Committee. Experiments were conducted in accordance with the National Institutes of Health Guide for the Care and Use of Laboratory Animals and reported in compliance with the ARRIVE guidelines (McGrath et al., 2010).

### Controlled Cortical Impact

Mice acclimatize to the animal facility for at least 1 week prior to experimentation. Male C57BL/6 mice were placed under 2–4% isoflurane (0.5 L/m) to shave the head and maintained under anesthesia using a nose cone once placed in a Kopf stereotactic frame. Using sterile technique of 70% alcohol and 10% betadyne were applied to the shaved head and the skull was exposed by a midline incision and skin retracted. Local lidocaine (2.5%) and prilocaine (2.5%) cream was administered to the incision site and a 3 mm diameter opening was made in the skull (+2 mm ML and −2 mm AP from Bregma). The bone was removed and the right cortex was exposed. The cortex was then compressed by using an electromagnetic Impact One device (Leica Biosystems, Buffalo Grove, IL) to deliver a 2 mm diameter rounded tip weight at a velocity of 3.5 m/s to a depth of 1 mm for a duration of 50 ms. The skin was placed back into position and closed with sterile wound clips and the mouse was placed on a heating pad until regaining consciousness before being placed back in the home cage. Sham mice went through the procedure except for delivery of impact.

### Brain Processing

Mice were anesthetized with urethane (4 g/kg; 0.2 ml; IP) and cardiac perfused with 0.1 M phosphate buffered saline (PBS) followed by 4% paraformaldehyde (PFA) in 0.1 M PBS. Brains were extracted, post fixed for 24 h in 4% PFA at 4°C, cryopreserved in 30% sucrose PBS solution, and frozen in optimal cutting temperature (OCT) compound on dry ice. Brains were stored at −80°C until cryosectioning. Twenty-four hours prior to cryosectioning, brains were acclimated overnight at −20°C and then cut with a Leica CM1950 cryostat at 30 μm-thick serial sections and stored in 0.1 M PBS + 0.02% sodium azide at 4°C as free-floating sections before processing for immunofluorescence and CS/DS-GAG extraction.

### Immunofluorescent Labeling and Confocal Microscopy

**Figure 1:**
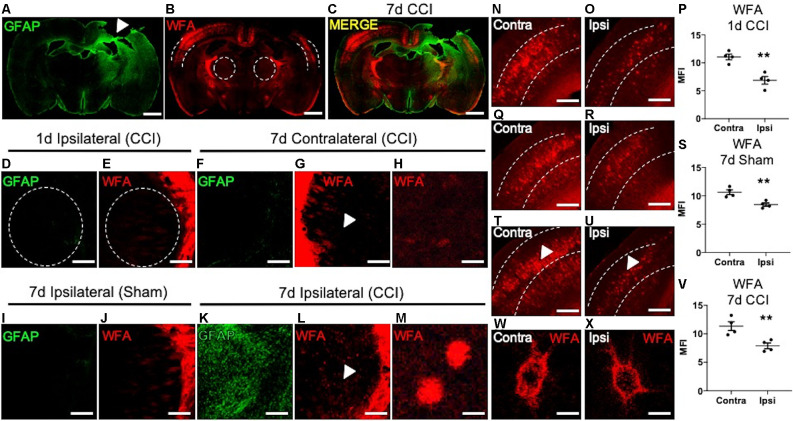
Loss of cortical PNNs and appearance of thalamic WFA^+^ puncta post-CCI in mice. A representative tile image of a coronal section (30 μm) from a C57BL/6 mouse brain at 7 days following an open-skull CCI procedure. Robust GFAP immunostaining is observed at the cortical injury site **(**white arrow, **A)**, which extends ventrally into the ipsilateral thalamus, but not the contralateral thalamus. Immunofluorescence detection of WFA, a lectin that associates with the CS/DS-GAGs of PNNs, was markedly decreased in the ipsilateral cortex (*dashed lines*) adjacent to the impact site **(B)**, and merged image **(C)**. At 1-day post-CCI, normal expression of GFAP **(D)** and WFA **(E)** were observed in the ipsilateral thalamus (*dashed lines*). Normal expression of GFAP **(F)** and WFA** (G)** were observed in the contralateral thalamus at 7 days post-CCI, white arrow in **(G)** demarcates magnified image of normal thalamic WFA expression **(H)**. The ipsilateral thalamus of 7- day post-sham control mice showed normal expression of GFAP **(I)** and WFA **(J)**. At 7 days post-CCI, the ipsilateral thalamus showed robust GFAP **(K)** and WFA immunostaining **(L)**. White arrows in **(L)** demarcate the appearance of WFA^+^ puncta in the ipsilateral thalamus at 7 days post-CCI **(M)**. Normal levels of WFA^+^ PNNs are observed in the contralateral cortex at 1-day post-CCI **(N)**, 7 days post-sham **(Q)**, and 7 days post-CCI **(T)**, a decrease in WFA^+^ PNNs were observed in the ipsilateral cortex at 1-day post-CCI **(O)**, 7 days post-sham **(R)**, and 7 days post-CCI **(U)**, and quantified in **(P,S)**, and **(V)**. PNN morphology of WFA immunolabeled PNN structures were similar between the contralateral **(W)** and the ipsilateral **(X)** cortices at 7 days post-CCI. **(P,S,V)** Student’s *t*-test, *N* = 4, **P < 0.01. Scale bars = 1,000 μm **(A–C)**, 200 μm **(D–G,I–L,N–U)** and 10 μm **(H,M,W,X)**.

Mouse 30 μm-thick serial coronal sections underwent antigen retrieval in 10 mM trisodium citrate (pH 8.5) and heated at 90°C for 20 m. Immunostaining was performed according to our previously published methods (Alonge et al., 2019, 2020). Briefly, free-floating tissue sections were permeabilized for 30 min at room temperature (RT) in 0.1 M PBS + 0.2% Triton X-100 and blocked for 2 h at 37°C in 0.1 M PBS + 0.05% Triton X-100 (PBST) + 10% normal donkey serum (Jackson ImmunoResearch). Sections were then incubated overnight at 4°C using 1:1,000 dilution of biotin-labeled *Wisteria floribunda* agglutinin (WFA; Sigma: L1516) in PBST + 1% donkey serum. Other primary antibodies used include: Iba1 (WAKO: 019-19741, RRID: AB_839504, 1:1,000), GFAP (Millipore: AB5541, RRID: AB_177521, 1:2,000), and UCHL1 (Cell Signaling: 13179, RRID: AB_2798141, 1:100). The following day, sections were washed and incubated for 2 h at RT in a 1:1,000 dilution of Alexa-fluor-conjugated secondary antibodies in PBST + 1% donkey serum. Sections were then counterstained for DAPI, mounted, and coverslipped using Fluoromount-G (ThermoFisher, 4958-02). Imaging was conducted with a Zeiss Axio Observer 7 inverted microscope with a 10× objective. Confocal microscopy was performed with a Nikon A1R HD confocal 20X objective. Images were processed with Fiji open-source imaging software. To quantify the mean fluorescent intensity (MFI) for each target we defined a set region of interest within the thalamus (see outlines in Figure 1C), converted the images to 8-bit, subtracted a constant background, and quantified the MFI normalized to the outlined area (per mm; Carulli et al., 2010) as previously described (Alonge et al., 2020). To calculate colocalization between two targets, we used the Coloc 2 plugin (Fiji) to measure the Pearson’s correlation coefficient (r value) from images that were processed for MFI quantification.

### RNAScope

Mouse 15 μm-thick coronal sections were prepared as above, mounted on microscope slides, and stored at −80°C for analysis. *In situ* hybridization for mouse, CHST3 mRNA was performed by RNAScope using protocols and reagents from the manufacturer (ACD, RNAScope Multiplex Fluorescent Reagent Kit v2). Briefly, sections were dehydrated through graded ethanols, treated with hydrogen peroxide to block endogenous peroxidases, followed by antigen retrieval using a preheated kit buffer for 15 m in a 95°C waterbath. After a brief rinse with distilled water and dehydration in ethanol, slides were treated with Protease III for 30 m at 40°C in a humid chamber, rinsed, and hybridized with MmCHST3probe (ACD, 572521) for 2 h at 40°C. Slides were stored overnight at RT in 5× SSC. The following day, slides were washed in RNAScope Wash Buffer and underwent three cycles of amplification at 40°C. The CHST3 signal was developed by incubation with HRP-C1 probe for 15 m at 40°C, followed by Opal570 substrate (Akoya Biosciences FP1488001KT, 1:1,500 in ACD Amplification Buffer) for 30 m at 40°C. Slides were washed, and residual peroxidase activity blocked with HRP Block (ACD kit). Slides were then blocked for 10 m at RT with Antibody Diluent/Block (Akoya ARD1001EA), incubated with mouse anti-GFAP (Covance smi-22, RRID: AB_509980, 1:1,000) for 1 h at RT, washed, and incubated with Opal polymer HRP secondary antibody (Akoya ARH1001EA) for 10 m at RT. Slides were then washed, developed with Opal520 substrate (Akoya FP1487001KT, 1:100 in 1× Plus Amplification Diluent, Akoya FP1498) for 10 m at RT. Slides were counterstained with DAPI, coverslips mounted, and imaged as above.

### CS/DS-GAG Digestion and Disaccharide Isolation

CS/DS disaccharides were isolated and quantified according to our previously published methods (Alonge et al., 2019, 2020; Logsdon et al., 2021). Five PFA-fixed coronal brain sections (30 μm) from each mouse were chosen for CS/DS disaccharide extraction and quantification. Sections were washed 3× in Optima LC/MS-grade water and 1× in 50 mM ammonium bicarbonate (pH 7.6) at RT. Chondroitinase ABC enzyme (ChABC, Sigma, C3667), a combinatorial endo- and exo-lyase that selectively degrades all CS/DS-GAG chains into their individual disaccharide units (Yamagata et al., 1968; Hamai et al., 1997; Prabhakar et al., 2009; Spliid et al., 2021), was reconstituted (500 mU/ml) in 50 mM ammonium bicarbonate (pH 7.6) to digest CS/DS-GAGs at 37°C in a Thermo Scientific MaxQ4000 orbital shaker for 24 h. Supernatants were collected in sterile 1.7 ml microcentrifuge tubes and spun for 10 m at 14k× *g* to pellet any debris. The supernatant was then dehydrated using a SpeedVac Concentrator, and the lyophilized product was reconstituted in 30 μl of LC/MS-grade water.

### LC-MS/MS + MRM Quantification of CS/DS Disaccharides

Isolated CS/DS samples were analyzed using a triple quadrupole mass spectrometer equipped with an electrospray ion source (Waters Xevo TQ-S) operated in negative mode ionization. LC-MS/MS was performed using a Waters Acquity I-class ultra-performance liquid chromatographic system (UPLC) coupled to the same Waters Xevo TQ-S system. Disaccharides were resolved by porous graphitic chromatography (Hypercarb column; 2.1 × 50 mm, 3 μm; ThermoFisher) as described previously (Alonge et al., 2019, 2020; Logsdon et al., 2021). Assigned MRM channels were: 4S-CS, *m/z* 458 > 300; 2S4S-DS and 4S6S-CS, *m/z* 538 > 300; 6S-CS, *m/z* 458 > 282; 2S6S-CS, *m/z* 268 > 282; 0S-CS, *m/z* 378 > 175. MassLynx software version 4.1 (Waters) was used to acquire and quantify all data. Under the conditions described above, the ratios between peak areas produced from equimolar CS standard runs were normalized to the highest peak intensity and relative quantification of each CS isomer within a sample was achieved using a modified peak area normalization function (Alonge et al., 2019). Each CS isomer was expressed as a relative abundance percentage of the total CS/DS isomer composition within a brain sample. Industry-grade CS disaccharide standards 0S-CS (OC28897), 4S-CS (OC28898), 6S-CS (OC01702), 4S6S-CS (OC28899) were purchased from Carbosynth (Berkshire, UK). The 2S4S-DS (CD005) and 2S6S-CS (CD006) were purchased from Galen Laboratory Supplies (North Haven, CT). Acetonitrile (optima LC/MS-grade) and all other reagents were obtained from Fisher Scientific.

### Rotarod

To determine whether changes in CS/DS-GAG composition correlated with TBI-induced motor dysfunction through disrupted thalamocortical circuitry (Wiley et al., 2016), a rotarod apparatus (San Diego Instruments, San Diego, CA) was used to measure motor coordination. For each of three trials, the rod gradually accelerated from 8, 16, 24, 32, and 40 rpm over a 120 s duration and remained at the final speed for an additional 30 s, if necessary. 5 m inter-trial intervals were used. The latency to fall off the rotarod was recorded for each trial. On the day of Rotarod training, each mouse was exposed to an Open Field for 5 m and distance traveled was measured to determine general physical activity.

### Open Field Test

An Open Field Apparatus (40 cm, Stoelting, Wood Dale, IL) was used to measure locomotor activity in each experimental group. Each mouse was allowed to freely explore the open field for 5 m and video recordings of the total distance traveled were analyzed using Anymaze software (Stoelting, Wood Dale, IL).

### Statistics

Sample sizes of *N* = 4/group were chosen, predicated on detecting ~80% power and alpha set at *P* < 0.05, with two tails. A two-sample unpaired Student’s *t*-test was used for two-group comparisons used GraphPad Prism^®^ 8.0 (Graph Pad Software, Inc., La Jolla, CA). Three or more samples were compared with a two-way analysis of variance (ANOVA). A Dunnett’s *post hoc* test was used to compare mice with surgery to a naïve control group. Error bars represent the standard deviation (SD) of the mean. Data met the normality assumptions of the statistical tests. Investigators were not blinded to injury conditions during euthanasia or histological preparation. Investigators were blinded to study conditions during quantitative LC-MS/MS and histological analyses.

## Results

### CCI Reveals Aberrant Perineuronal Net Morphology Associates With Thalamocortical Circuitry

PNN matrix detection is accomplished using immunofluorescence labeling of the PNN-associated CS/DS-GAGs using *Wisteria floribunda* agglutinin (WFA) in mice (Alonge et al., 2020). We examined regional changes in PNN matrix integrity in association with CCI-induced gliosis at 1 and 7 days post-CCI. We observed an association between induced astrogliosis (GFAP, Figure 1A) and loss of cortical PNNs (WFA, Figure 1B) at 7 days post-CCI in coronal sections at Bregma −1.0 mm between the ipsilateral and contralateral hemispheres from mice. These histological changes radiated from the injury site along the dorsolateral layers of the cortex and into the ipsilateral thalamus (Figure 1C). At 1 day post-CCI we observed low levels of GFAP and WFA labeling in the ipsilateral thalamus (Figures 1D,E). At 7 days post-CCI, the contralateral CCI thalamus (Figures 1F–H) and ipsilateral sham control thalamus (Figures 1I,J) also exhibited low levels of GFAP and WFA labeling (Figure 1J). However, a close examination of the ipsilateral thalamus of 7 days post-CCI mice revealed the appearance of robust astrogliosis (GFAP, Figure 1K) and WFA^+^ puncta (Figures 1L,M). These spatiotemporal changes in thalamic WFA^+^ puncta are in contrast with changes observed in cortical PNNs, which showed a strong reduction in WFA^+^ PNNs after 1 day CCI (Figures 1N–P, *P = 0.0026*), 7 day Sham (Figures 1Q–S, *P = 0.0056*), and 7 day CCI (Figures 1T–V, *P = 0.0094*). Unlike the thalamic WFA^+^ structures, cortical PNN morphology is comprised of dense, extracellular honeycomb-like structures, with similar net morphology seen in both uninjured contralateral (Figure 1W) and injured ipsilateral (Figure 1X) cortices at 7 days post-CCI. Although we observe normal WFA^+^ PNN morphology in both the injured and uninjured cortices at 7 days post-CCI, thalamic WFA^+^ puncta appeared only after 7 days post-CCI (Figure 1M).

Changes in WFA labeling may indicate a shift in the composition of CS/DS-GAG chains that often associate with PNN disintegrity, reminiscent of a plastic environment within the developing brain. During neurodevelopment, reduced WFA^+^ PNN abundance is associated with an increase in the relative abundance of the 6S-CS isomer (Carulli et al., 2010). Moreover, elevated 6S-CS isomer overexpression delays PNN maturation (Miyata et al., 2012) and also associates with proteolytic degradation of PNN matrices in the mouse brain (Miyata and Kitagawa, 2016). Although increased 6S-CS (Properzi et al., 2005) and PNN loss (Wiley et al., 2016) have been reported in mice after CCI, the biochemical mechanisms associated with PNN integrity are poorly understood. Therefore, we sought to explore whether CCI induces changes in CS/DS-GAG sulfation patterns in association with PNN loss, gliosis, and impaired motor coordination in mice with TBI.

### Spatiotemporal Dynamics of CS/DS-GAG Sulfation in the Ipsilateral Thalamus After CCI

Using LC-MS/MS to quantitatively analyze the relative abundance of CS/DS isomers from a series of fixed-brain coronal sections expanding the rostro caudal extent of the injury site (−1.0 to −3.0 mm AP from the Bregma; Alonge et al., 2019), we found robust changes in brain CS/DS-GAG sulfation patterns 7 days post-CCI, including hypersulfation (*P < 0.0001*) of CS/DS-GAGs by increased 6S-CS (*P < 0.0001*) and 2S6S-CS (*P = 0.0001*) isomers and decreased 0S-CS (*P < 0.0001*) and 2S4S-DS (*P = 0.02*) isomers, compared to naïve controls (Table 1). We also found modest changes in brain CS/DS-GAG sulfation at 7 days in sham mice compared to naïve control mice, implying the act of skull removal alone to be sufficient to induce lesser changes in brain CS/DS-GAG sulfation. As such, the degree of changes observed in brain CS/DS-GAG sulfation patterns can be highlighted as a potential indicator of injury severity.

**Table 1:** Relative percentages of CS isomers in the means of five representative coronal sections spanning the rostro-caudal extent of the injury site (−1.0, −1.5, −2.0, −2.5, and −3.0 mm from Bregma).

%CS/DS (SD)	Naïve (*n* = 4)	7d Sham (*n* = 4)	1d CCI (*n* = 4)	7d CCI (*n* = 4)	*F Value*	*P Value*
Δ0S-CS	7.48 (0.42)	6.19*** (0.31)	7.45 (0.19)	4.68*** (0.39)	*F*_(3, 12)_ = 60.63	<0.0001
Δ4S-CS	84.66 (0.66)	85.49 (0.95)	84.37 (0.44)	85.02 (0.75)	*F*_(3, 12)_ = 1.79	0.203
Δ6S-CS	3.53 (0.07)	3.85 (0.34)	3.70 (0.05)	4.94*** (0.31)	*F*_(3, 12)_ = 29.55	<0.0001
Δ2S6S-CS	1.89 (0.08)	2.52* (0.31)	1.86 (0.37)	3.26*** (0.42)	*F*_(3, 12)_ = 16.90	0.0001
Δ4S6S-CS	1.57 (0.08)	0.77*** (0.14)	1.82 (0.24)	0.83*** (0.19)	*F*_(3, 12)_ = 36.37	<0.0001
Δ2S4S-DS	0.87 (0.17)	1.19 (0.27)	0.80 (0.26)	1.28* (0.11)	*F*_(3, 12)_ = 4.82	0.0200
Avg #S PerΔCS	0.97 (0.003)	0.98* (0.005)	0.97 (0.007)	1.01*** (0.007)	*F*_(3, 12)_ = 35.47	<0.0001

CS, Chondroitin Sulfate; DS, Dermatan Sulfate; CCI, Controlled Cortical Impact; One-way ANOVA *post hoc* Dunnett’s; *P ≤ 0.05, ***P ≤ 0.001 vs. Naïve. Data are shown as mean ± SD.

We then predicted that the robust appearance of WFA^+^ puncta in the gliotic thalamus 7 days post-CCI (Figure 1) would also associate with changes in CS/DS-GAG sulfation patterns and contribute to the whole brain CS/DS-GAG changes as described in Table 1. In the second cohort of mice, we conducted interregional LC-MS/MS to measure CS/DS-GAG sulfation changes specifically in the ipsilateral thalamic region in naïve, sham, and CCI mice 7 days post-CCI. We found a significant increase in thalamic 6S-CS isomer (*P = 0.002*) and a trend for increased 2S6S-CS isomer (*P = 0.07*) 7 days post-CCI, and to a lesser extent at 7 days after sham, when compared to naïve controls (Table 2). Although changes in CS/DS-GAG total abundance have been reported in rodents exposed to TBI (Properzi et al., 2005), our work builds upon these initial discoveries by describing the regional and temporal changes in CS/DS-GAG sulfation patterns present in the progression of CCI/TBI injury.

**Table 2:** Relative percentages of CS isomers in the means of 10 representative thalamic isolations spanning the rostro-caudal extent of the injury site (−1.0, −1.5, −2.0, −2.5, and −3.0 mm from the Bregma) of mice used in the rotarod task.

%CS/DS (SD)	Naïve (*n* = 4)	7d Sham (*n* = 4)	7d CCI (*n* = 4)	*F Value*	*P Value*
Δ0S-CS	10.57 (1.17)	10.74 (3.48)	8.52 (1.40)	*F*_(2, 9)_ = 1.18	0.35
Δ4S-CS	78.76 (2.31)	76.61 (2.47)	77.77 (3.34)	*F*_(2, 9)_ = 0.61	0.56
Δ6S-CS	3.55 (0.44)	4.29* (0.29)	5.04*** (0.45)	*F*_(2, 9)_ = 13.80	0.002
Δ2S6S-CS	4.91 (1.17)	6.62 (0.69)	6.46 (1.12)	*F*_(2, 9)_ = 3.47	0.077
Δ4S6S-CS	1.13 (0.15)	0.79 (0.32)	0.75 (0.22)	*F*_(2, 9)_ = 3.09	0.095
Δ2S4S-DS	1.08 (0.13)	0.99 (0.15)	1.41 (0.49)	*F*_(2, 9)_ = 2.14	0.17
Avg # S Per ΔCS	0.97 (0.01)	0.98 (0.04)	1.001 (0.01)	*F*_(2, 9)_ = 2.47	0.14

CS, Chondroitin Sulfate; DS, Dermatan Sulfate; CCI, Controlled Cortical Impact; One-way ANOVA *post hoc* Dunnett’s; *P ≤ 0.05, ***P ≤ 0.001 vs. Naïve. Data are shown as mean ± SD.

### The Appearance of Thalamic WFA^+^ Puncta Colocalizes with UCHL1^+^ Neurons After CCI

**Figure 2:**
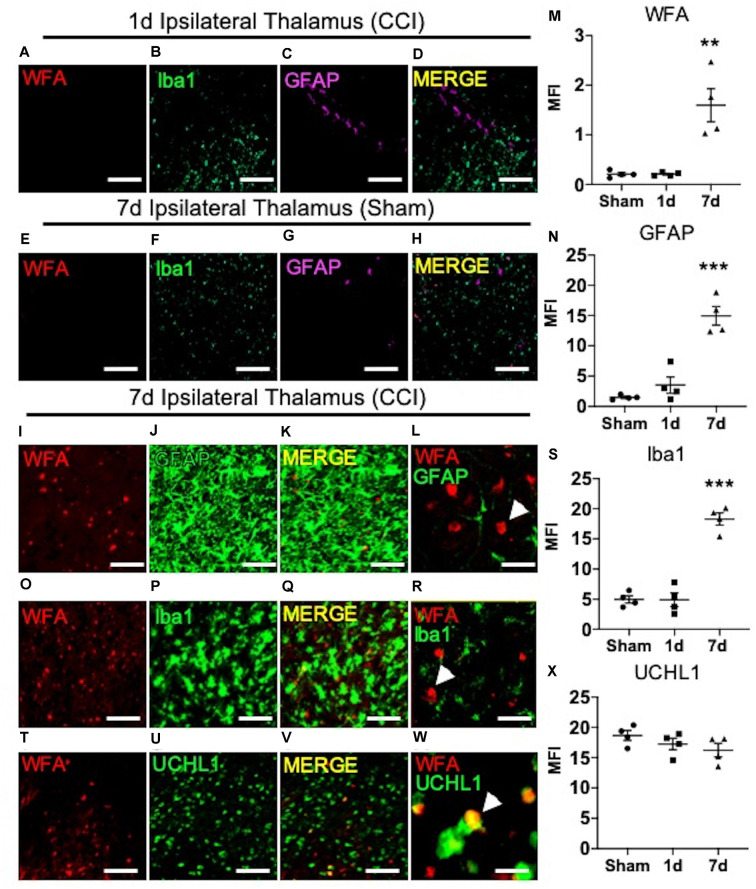
The appearance of thalamic WFA^+^ puncta moderately associates with UCHL1^+^ neurons 7 days post-CCI. Representative images of the ipsilateral thalamus at 1 day post-CCI show normal expression of WFA **(A)**, Iba1 **(B)**, GFAP **(C)**, and merged image **(D)**. Representative images of the ipsilateral thalamus at 7 days after the sham procedure show normal expression of WFA **(E)**, Iba1 **(F)**, GFAP** (G)**, and merged images **(H)**. The appearance of ipsilateral thalamic WFA^+^ puncta **(I,O,T)** occurred in association with astrogliosis (GFAP, **J**; merged image **K,L**; quantified in **M** and **N**), microgliosis (Iba1, **P**; merged image **Q,R**; quantified in **S**), and UCHL1^+^ neurons (**U**; merged image **V,W**; quantified in **X**). High magnification image shows that GFAP **(L)**, Iba1 **(R)**, and UCHL1 **(W)** weakly colocalized with WFA^+^ puncta in the thalamus (*white arrow demarcates WFA^+^ puncta*). One-way ANOVA Dunnett’s *post hoc* test **(M,N,S,X)**. Values represent means ± SD (*n* = 4, **P < 0.01; ***P < 0.001). Scale bars = 200 μm **(A–K,O–Q,T–V)** and 20 μm **(L,R,W)**. WFA, *wisteria floribunda* agglutinin; UCHL1, ubiquitin C-terminal hydrolase L1; MFI, mean fluorescent intensity; CCI, controlled cortical impact; GFAP, Glial fibrillary acidic protein.

Previous reports describe a well-established peak in gliosis occurring between 1 and 7 days after CCI/TBI, which can persist depending on injury severity (Burda and Sofroniew, 2014). Based on these previous reports, we next predicted that the spatiotemporal appearance of ipsilateral thalamic WFA^+^ puncta (Figure 1M) may associate with temporal expression of glial scarring (Figure 1K) within the ipsilateral thalamus. As expected, based on our initial findings described in Figure 1, we did not observe a robust appearance of WFA^+^ puncta (Figure 2A), microgliosis (Iba1, Figure 2B), or astrogliosis (GFAP, Figure 2C) in the ipsilateral thalamus at 1-day post-CCI (Figure 2D), which were also absent in the ipsilateral thalamus 7 days post-sham (Figures 2E–H). However, we observed a robust appearance of WFA^+^ puncta (Figure 2I; quantified in Figure 2M, *P = 0.0012*), astrogliosis (GFAP, Figure 2J; quantified in Figure 2N, *P < 0.0001*), and microgliosis (Iba1, Figure 2P; quantified in Figure 2S, *P < 0.0001*) in the ipsilateral thalamus 7 days post-CCI. Surprisingly, thalamic WFA^+^ puncta showed weak colocalization with astrogliosis (Figures 2I–L; Pearson’s *r* = 0.11) and microgliosis (Figures 2O–R, Pearson’s *r* = 0.13) within this region, suggesting the production of WFA^+^ puncta may be present in another CNS cell type.

In addition to GFAP, patients with TBI also exhibit increased biofluid expression of the clinical biomarker of neuronal repair, ubiquitin C-terminal hydrolase L1 (UCHL1; Brophy et al., 2011; Wang et al., 2021). Since thalamic WFA^+^ puncta is only weakly associated with astro- and micro-gliosis, we next examined the level of colocalization between thalamic WFA^+^ puncta and UCHL1^+^ neurons 7 days post-CCI injury. Interestingly, although protein expression of UCHL1 remained unchanged in 7-days post-sham, 1-days post-CCI, and 7-days post-CCI mice (Figure 2U; quantified in Figure 2X, *P = 0.26*), we discovered a moderate colocalization of UCHL1 with WFA^+^ puncta within this region (Figures 2T–W; Pearson’s *r* = 0.47), implying that the appearance of WFA^+^ puncta associates with neuronal repair, rather than gliosis, after CCI injury.

### CHST3 Is Elevated Within the Thalamocortical Circuit Following CCI

**Figure 3:**
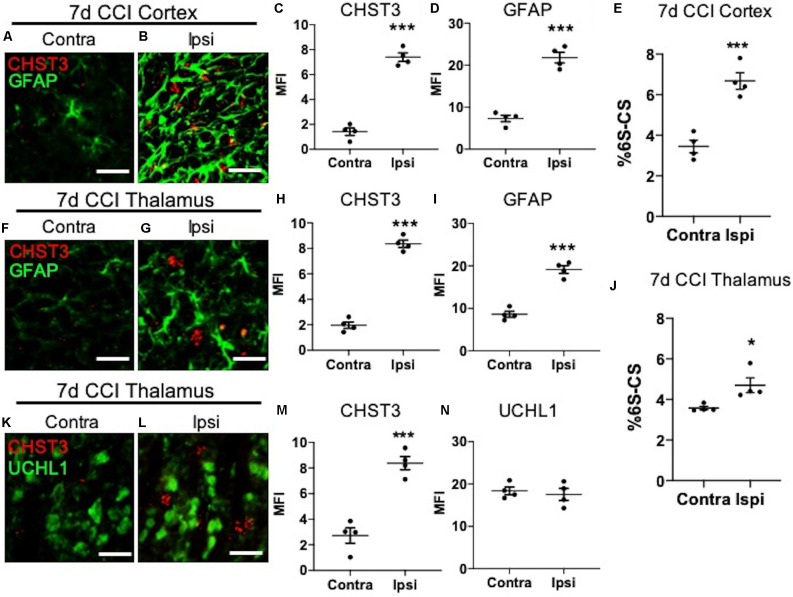
Elevation of CHST3 expression and 6S-CS isomer abundance at the site of cortical injury and ipsilateral thalamus at 7 days post-CCI. Representative images of RNAscope analysis paired with immunofluorescent analysis showed low expression of the 6S-CS-specific sulfotransferase, CHST3, and GFAP in the contralateral cortex **(A)** relative to the ipsilateral cortex **(B)** at 7 days post-CCI, and quantified in **(C)** and **(D)**. LC-MS/MS analysis found an increase in the relative abundance of ipsilateral 6S-CS compared to the contralateral cortex **(E)**. Low expression of CHST3 and GFAP was observed in the contralateral thalamus **(F)** compared to the ipsilateral thalamus **(G)** 7 days post-CCI, and quantified in **(H)** and **(I)**. A significant increase in the relative abundance of 6S-CS was measured in the ipsilateral thalamus **(J)**, compared to the contralateral thalamus 7 days post-CCI. Low expression of CHST3, yet normal expression of UCHL1, was observed in the contralateral thalamus **(K)** compared to the ipsilateral thalamus **(L)** 7 days post-CCI, and quantified in **(M)** and **(N)**. **(C–E,H–J,M,N)** Student’s *t*-test. Values represent means ± SD (*N* = 4, *P < 0.05; ***P < 0.001). Scale bars = 20 μm. CHST3, carbohydrate sulfotransferase 3; CCI, controlled cortical impact; UCHL1, ubiquitin C-terminal hydrolase L1; GFAP, Glial fibrillary acidic protein.

We next sought to determine the cellular source for increased levels of the 6S-CS isomer by measuring the expression of the 6S-CS-specific sulfotransferase (CHST3) in mice 7 days post-CCI exposure. Since the induction of astrogliosis appeared in association with increased levels of the 6S-CS isomer, we combined immunohistochemistry with RNA scope to determine whether CHST3 mRNA colocalized with GFAP immunofluorescence. As expected, we observed an increase in ipsilateral cortical CHST3 (*P < 0.0001*) and GFAP (*P < 0.0001*; Figures 3A,B; quantified in Figures 3C,D) 7 days post-CCI, complementing an increase in the 6S-CS isomer (*P = 0.0007*; Figure 3E) within this region compared to the uninjured contralateral cortex. This finding was recapitulated in the ipsilateral thalamus 7 days post-CCI, which also showed increased CHST3 (*P < 0.0001*) and GFAP (*P < 0.0001*; Figures 3F,G; quantified in Figures 3H,I) in association with increased thalamic 6S-CS isomer (*P = 0.0243*; Figure 3J) compared to the uninjured contralateral thalamus. Despite previous reports of an association between CHST3 and injury-induced GFAP (Properzi et al., 2005), CHST3 expression weakly colocalized with GFAP immunoreactivity in both the cortex (Pearson’s *r* = 0.22) and thalamus (Pearson’s *r* = 0.24).

The lack of a strong association between CHST3 and GFAP suggests additional cell type(s) may be responsible for the increase in 6S-CS sulfation after CCI. We predicted that the increase in thalamic 6S-CS may be linked to the specific appearance of thalamic WFA^+^/UCHL1^+^ cells (Figure 2W). Surprisingly, RNAscope analysis found that CCI-induced thalamic ipsilateral CHST3 expression (*P = 0.0004*) only weakly colocalized with UCHL1 immunoreactivity (Figures 3K,L; quantified in Figures 3M,N; Pearson’s *r* = 0.10) in the thalamus. Again, no difference in the thalamic expression of UCHL1 was observed at 7 days post-CCI (Figure 3N), agreeing with our previous finding (Figure 2X). The stronger association between UCHL1 and WFA^+^ puncta (Figure 2W) compared to UCHL1 and CHST3 (Figure 3L) implies the appearance of CCI-induced ipsilateral thalamic WFA^+^ puncta may not be strongly related to the increased levels of 6S-CS sulfation detected after CCI.

### CCI-Induced Increase in the Thalamic 6S-CS Isomer Associates With Motor Coordination Deficits

**Figure 4:**
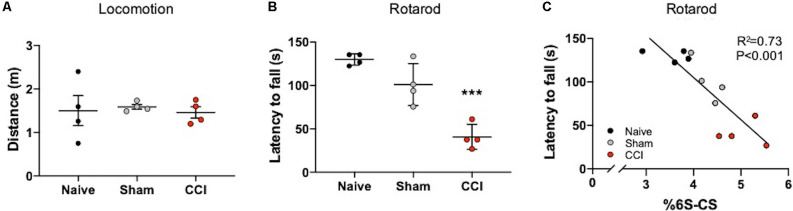
Motor coordination deficits correlate with the abundance of thalamic 6S-CS after cortical brain injury. No differences in locomotion were measured in mice subject to the Open Field Task at 7 days post-CCI, or sham procedure, compared to naïve control mice **(A)**. Rotarod testing revealed significant motor coordination deficits in CCI mice as evidenced by a reduced latency to fall from the apparatus **(B)**. The relative abundance of 6S-CS in the ipsilateral thalamus was significantly correlated to latency **(C)**. **(A,B)** One-way ANOVA Dunnett’s *post hoc* test. **(C)** Linear regression. Values represent means ± SD (*n* = 4, ***P < 0.001).

Mice with TBI are known to develop motor coordination deficits (Meabon et al., 2016; Bhowmick et al., 2018) that can be assessed using a rotarod task (Logsdon et al., 2020). In order to specifically address deficits in motor coordination with the rotarod task, any confounds of general locomotion impairment were addressed using the Open Field Task. Mice exposed to CCI and sham procedures did not exhibit locomotor deficits 7 days post-procedures compared to naïve mice, as evidenced by similar distances traveled in the Open field task (Figure 4A). In contrast, a significant decrease in rotarod latency was observed in mice at 7 days post-CCI compared to naïve control mice and, to a lesser extent, sham mice (Figure 4B, *P < 0.0001*), which demonstrates severe motor impairment selectively in these CCI-exposed mice.

The thalamus is an important brain region involved in relaying information to the cortex for motor function (Biane et al., 2016; Tanaka et al., 2018). In our model system, the cortex is mostly removed from this circuit, yet the thalamus remains intact (Figure 1A). Since 6S-CS sulfation is required for neuroplasticity and associated behavioral deficits in mice (Yang et al., 2021), we sought to determine whether the significant differences in thalamic 6S-CS abundance (Table 2) were associated with motor coordination defects in mice with CCI. As such, we correlated the latency to fall with the thalamic 6S-CS levels for naïve, 7 days post-sham, and 7 days post-CCI mice. Here, we found a strong correlation between the abundance of thalamic 6S-CS isomer and rotarod performance (Figure 4C; *R*^2^ = 0.73, *P < 0.001*). These findings provide a functional association between thalamic CS/DS-GAG sulfation and motor coordination deficits, warranting future mechanistic studies targeting the 6S-CS isomer in preclinical models for human TBI.

## Discussion

Glial scar formation, driven primarily by CSPG and associated sulfated CS/DS-GAG secretion into the extracellular space (Silver and Miller, 2004), influences axonal regeneration after CNS injury (Bradbury et al., 2002; Anderson et al., 2016). Sulfation patterns of the CS/DS-GAG chains influence cellular interactions with specific growth factors capable of attracting or repelling axonal growth (Djerbal et al., 2017). The balance between circuit formation and maturation is best observed during normative aging, where the aging mouse brain shows a steady decrease in the relative abundance of the neuroplastic 6S-CS isomer in the mouse brain (Foscarin et al., 2017) that results in restricted circuit plasticity and enhanced synapse stability. In contrast to normative aging, previous work has shown a paradoxical *increase* in 6S-CS isomer expression in response to TBI-induced glial scarring (Properzi et al., 2005). Here, we expand upon these initial reports to show CCI induces distinct spatiotemporal changes in the brain CS/DS-GAG sulfation patterns that associate with the development of glial scarring, neuronal UCHL1 expression, and well-established CCI-induced motor behavioral deficiencies.

Using LC-MS/MS, we examined changes in whole brain (e.g., coronal sections) and interregional (e.g., cortical vs. thalamic) CS/DS-GAG sulfation patterns in mice at 1 and 7 days following a single CCI exposure. We found that severe glial scar formation at the site of cortical impact 7 days post-CCI associated with histological detection of reactive astrocytes and microglia, as well as a robust increase in CHST3 mRNA expression and corresponding 6S-CS isomer abundance. Paralleling these changes, the ipsilateral thalamus also showed moderate reactive gliosis, elevated CHST3 expression, and 6S-CS abundance at 7 days post-CCI. The potential translational importance of these findings is evidenced by our findings that mice exhibiting the highest abundance in thalamic 6S-CS also demonstrated the worst motor performance on the rotarod task. One limitation of this study is the low number of mice used in each experimental group. Because we observed profound differences in CS/DS-GAG sulfation patterns, thalamic gliosis, and motor deficits at 7 days after CCI, we proceeded with our report in order to reduce the number of mice we needed to expose to severe brain injury. Taken together, our results indicate that thalamic 6S-CS production may be related to TBI severity and contribute to impaired motor coordination through disruptions in the thalamocortical network.

The loss of stable PNN structures in the ipsilateral cortex (Figure 1) in association with elevated 6S-CS isomer abundance (Figure 3) warrants further discussion. Since elevated 6S-CS abundance drives neurocircuit reorganization through mature PNN destabilization (Miyata and Kitagawa, 2016), we first predicted that TBI-induced loss of cortical PNNs could result from the upregulation in astroglia-associated 6S-CS isomer production (Properzi et al., 2005). Surprisingly, we observed a weak colocalization of CHST3 mRNA with either GFAP^+^ astrocytes, Iba1^+^ microglia, or UCHL1^+^ neurons 7 days post-CCI. These results suggest the presence of another CNS cell type responsible for the upregulation of 6S-CS isomer after CCI and future work is warranted to identify these additional players.

Although the identity of the CHST3 expression cells remains elusive, we did observe the appearance of irregular WFA^+^ puncta throughout the ipsilateral thalamic region 7 days post-CCI. Rather than strongly associating with reactive gliosis, however, these unique WFA^+^ puncta exhibited a moderate colocalization with UCHL1^+^ neurons. UCHL1 activity has been shown to protect neurons from hypoxic injury by preserving excitatory synaptic drive to pyramidal neurons (Liu et al., 2019). As such, the WFA^+^ puncta associated with thalamic UCHL1 neurons may act as a protective barrier against CCI-induced neuroinflammation, indicating a mechanism of functional recovery and neuronal repair in the thalamocortical network post-injury. The morphology differences observed between cortical and thalamic WFA^+^ ECM structures are also of notable interest. PNN morphology is heterogenous throughout different brain regions; whereas PNN matrices within the visual cortex are highly structured (Alonge et al., 2020), hypothalamic PNNs appear more diffuse (Alonge et al., 2020). Here, we also observed similar differences between the intricate WFA^+^ PNN labeling in the motor and somatosensory cortices and the diffuse WFA^+^ structures in the thalamus (Figure 1). Such morphology differences may be influenced by the availability of extracellular scaffolding proteins that stabilize the PNN matrix, including Tenascin R and Hyaluronan and Proteoglycan Link Proteins (HAPLNs), which also appear to be brain region specific (Alonge et al., 2020). The cerebellum has also been implicated in motor coordination (Lee et al., 2015; Meabon et al., 2016; Logsdon et al., 2020), however, we did not observe significant changes in cerebellar ECM appearances, CS/DS-GAG composition, or induction of gliosis after CCI (data not shown). Regardless of PNN morphology, the enriched CS/DS-GAG chains function to interact with extracellular protein factors dependent on the CS/DS sulfation patterns (Kwok et al., 2011). For example, neurite extension is enhanced through interactions between 2S6S-CS and axonal guidance proteins (Shida et al., 2019).

In summary, mice exposed to CCI-induced TBI exhibit marked shifts in CS/DS-GAG sulfation in the ipsilateral site of brain injury. Here, we provide evidence to support a time-dependent association between reactive gliosis, appearance of injury-induced thalamic WFA^+^ puncta, and increased 6S-CS isomer expression 7 days after CCI-injury. The level of glial scar formation at the cortical site of injury and ipsilateral thalamus corresponded to the magnitude of 6S-CS and CHST3 expression, and that the increase in 6S-CS isomer abundance also tightly correlated to functional impairment in motor coordination. Although thalamic WFA^+^ puncta post-injury colocalized weakly with reactive astro- or micro-gliosis, their appearance did associate moderately with protective UCHL1^+^ neurons. Due to previous reports establishing UCHL1 as a validated biofluid biomarker for TBI severity (Brophy et al., 2011; Wang et al., 2021), and that UCHL1 protects neurons from injury-induced cell death (Liu et al., 2019), more research investigating the biological significance for the formation of WFA^+^ puncta associating with UCHL1^+^ neurons after CCI is thus warranted.

## Data Availability Statement

The raw data supporting the conclusions of this article will be made available by the authors, without undue reservation.

## Ethics Statement

The animal study was reviewed and approved by Veterans Affairs Puget Sound Health Care System Institutional Animal Care and Use Committee.

## Author Contributions

KA, MH, MY, DC, WB, and AL contributed to experimental design, data interpretation, and manuscript preparation. CCI and rotarod procedures were conducted by AL. Tissue sectioning and immunohistochemistry analyses were completed by MH and MY. Glycan isolations and mass spectrometry analyses were performed and evaluated by KA, MH, and AL. All authors contributed to the article and approved the submitted version.

## Conflict of Interest

The authors declare that the research was conducted in the absence of any commercial or financial relationships that could be construed as a potential conflict of interest.

## Publisher’s Note

All claims expressed in this article are solely those of the authors and do not necessarily represent those of their affiliated organizations, or those of the publisher, the editors and the reviewers. Any product that may be evaluated in this article, or claim that may be made by its manufacturer, is not guaranteed or endorsed by the publisher.
